# Functional diversity of the above-ground fungal community under long-term integrated, organic and biodynamic Vineyard Management

**DOI:** 10.1186/s40793-024-00625-x

**Published:** 2024-11-11

**Authors:** Katharina Steng, Friederike Roy, Harald Kellner, Julia Moll, Susanne Tittmann, Johanna Frotscher, Johanna Döring

**Affiliations:** 1 Department of General and Organic Viticulture, Hochschule Geisenheim University, Von-Lade- Str. 1, 65366 Geisenheim, Germany; 2 Department of Bio- and Environmental Sciences, TU Dresden, International Institute Zittau, Markt 23, 02763 Zittau, Germany; 3 Department of Soil Ecology, Helmholtz Centre for Environmental Research GmbH - UFZ, Theodor-Lieser-Str. 4, 06120 Halle (Saale), Germany; 4 Department of Plant Breeding, Hochschule Geisenheim University, Von-Lade-Str. 1, 65366 Geisenheim, Germany

**Keywords:** Management systems, Mycobiome, Microorganisms, Grapevine, *Vitis vinifera*

## Abstract

**Background:**

Sustainable agriculture increasingly emphasizes the importance of microbial communities in influencing plant health and productivity. In viticulture, understanding the impact of management practices on fungal communities is critical, given their role in disease dynamics, grape and wine quality. This study investigates the effects of integrated, organic, and biodynamic management practices on the diversity and function of fungal communities in a vineyard located in Geisenheim, Germany, focusing on above-ground parts such as bark, leaves, and grapes.

**Results:**

Our findings indicate that while overall fungal species richness did not significantly differ among management systems across various compartments, the composition of these communities was distinctly influenced by the type of management system. In particular, leaf and grape compartments showed notable variations in fungal community structure between integrated and organic/biodynamic management. No differences were observed between organic and biodynamic management. Integrated management demonstrated a significantly higher abundance of mycoparasites in comparison to organic and biodynamic management, primarily attributed to the increased presence of *Sporobolomyces roseus*, *Sporobolomyces ellipsoideus* and *Rhodotorula glutinis*.

**Conclusions:**

The findings highlight the importance of management practices in shaping fungal community composition and function in vineyards. Although overall species richness remained unaffected, community composition and functional diversity varied, highlighting the potential for strategic microbiome management to enhance vineyard sustainability and plant health.

**Supplementary Information:**

The online version contains supplementary material available at 10.1186/s40793-024-00625-x.

## Background

Agriculture has consistently faced challenges, particularly with its historical emphasis on maximizing yields and suppressing diseases, often at the expense of sustainability and biodiversity [1]. As the agricultural sector continues to address these challenges, recent decades have marked a significant shift in research: from focusing on traditional, tangible metrics like plant growth and yield optimization to investigating the complex microorganisms’ roles within agricultural ecosystems.

For a long lapse of time, farmers and researchers focused on microorganisms as biotic stressors causing diseases that needed to be eliminated. However, over the past century it could be shown that only a small proportion of microorganisms are associated with disease or pathogenicity. The majority of microbes play crucial rules in ecosystem functioning and are known for beneficial interactions with each other as well as with the plant [2]. Plants host a highly diverse variety of microorganisms and among these inhabitants, beneficial, neutral, and pathogenic microorganisms are present [3–5], and their interactions have diverse consequences for population dynamics, functional capacities within the microbiome, and plant performance [2]. While extensive research has been dedicated to understanding the composition and dynamics of bacterial constituents within the grapevine microbiota [6–9], there is comparatively less knowledge regarding other microbiota members, such as fungi [10, 11]. The broader plant mycobiome and its interactions have often been overlooked in grapevine microbiome research. Nevertheless, the crucial role of specific fungi in plant fitness is well recognized [12]. Pathogenic fungi can have a direct negative influence on plant health status, as their interaction can lead to a disease scenario, resulting in compromising the normal physiology and vitality of the plant [4]. Beneficial fungi and their interactions with other microbes and the plant can lead to an antagonistic effect against pathogens, thereby promoting plant health and performance [13]. Pathogen suppression can be based on different modes of action such as direct competition for space and nutrients, production of bioactive substances to inhibit microbial growth, namely antibiotics, antimicrobial compounds, lytic enzymes, or siderophores, or through induction of resistance [14–17]. Interactions between the plant and neutral or beneficial fungi show a wide range of indirect effects on plant performance, involving crucial functions such as plant nutrition and plant resistance to biotic and abiotic stresses, hence plant growth promotion, fruit yield, and survival [13, 18, 19]. It is now widely accepted that the microbiome and its balanced microbial composition are crucial factors for plant vitality [20].

Viticulture, characterized by its high input of pesticides and intensive management practices [21, 22], represents a critical area for exploring how microbial communities are impacted by human interventions, particularly given the crop’s economic importance and its vulnerability to a range of biotic and abiotic stresses. By examining how specific agricultural decisions affect a grapevine’s microbiome, we can potentially uncover strategies for leveraging beneficial microbial interactions to improve vineyard resilience and productivity. Previous research has highlighted various factors influencing microbial community structure. These factors encompass plant-related aspects such as genotype, age, and phenology, alongside edaphic factors like soil moisture, pH, and nutrient content. Additionally, environmental parameters such as temperature, radiation, and precipitation have been identified as significant contributors [4, 6, 23, 24]. Furthermore, different crop management practices have been demonstrated to induce alterations in microbial community composition [25–29].

The most common management systems in viticulture are integrated, organic, and biodynamic practices. Integrated cultivation is the most widespread practice in viticulture, even though there has been an increasing trend towards organic and biodynamic systems over the last 15 years [30]. The guidelines for integrated viticulture aim to integrate natural regulation processes with anthropic measures to be able to reduce external inputs. Practices like the use of mineral fertilizers, synthetic herbicides, and fungicides are allowed, following the conceptual framework of integrated pest management, which includes careful monitoring and preventive cultural practices [31, 32]. Organic viticulture is codified by the Council of the European Union (EC 2018/848; EC 2021/1165) as a system that supports agro-ecosystem health, including biodiversity, biological cycles, and soil biological activity [33, 34]. In comparison to integrated management, synthetic pesticides are prohibited and instead products of natural origin, such as copper, sulfur, and plant strengtheners are used. Furthermore, the application of synthetic mineral fertilizers and herbicides is forbidden. Mechanical weeding, organic fertilizers, green manure, and species-rich cover crop mixtures are intended to increase biodiversity and promote soil fertility and structure [25, 35, 36]. Biodynamic viticulture must follow the same regulations as organic viticulture, but uses specific preparations with the intention to stimulate vitalizing and harmonizing processes in the soil and the plant [34, 37]. The practices are based on Rudolf Steiner’s Agriculture Course, reflecting the anthroposophical and metaphysical concepts proposed by the founder [38].

The investigation of effects of differing management practices on the vineyard microbiome is increasingly pertinent. As the European Green Deal aims to expand organically farmed areas to 25% by 2030, it becomes imperative to delve deeper into various management regimes for more nuanced comparisons and insights into their impact on the ecosystem [39]. While previous research has explored the influence of viticultural management on microbial richness, activity, and composition, particularly in soil [25–29], the focus has predominantly been on structural rather than on functional differences [40]. There is a growing consensus that understanding the complex functions of these microbial communities is crucial for a comprehensive view of their impact on ecosystem functioning [41]. Therefore, integrating functional predictions with fungal profiling is essential for advancing our understanding of vineyard ecology and improving viticultural practices. Thus, this study seeks to fill this gap by investigating how management systems influence fungal abundance, unraveling potential shifts in community composition, and linking these differences to changes in functional groups within the prominent taxa in above-ground compartments such as bark, grapes, and leaves by amplicon sequencing.

To address these objectives, several research questions are posed: What effects did different viticultural management systems, namely integrated, organic, and biodynamic approaches, have on the above-ground fungal richness, and what variation exists among these systems? Do these systems exert distinct effects on fungal communities in different compartments, such as bark, leaf, and grape? Additionally, the study aims to identify characteristic fungal taxa associated with integrated, organic, or biodynamic management, and examine how these relate to functional groups. The overarching aim of this study is to generate insights that could inform strategic manipulation of the microbiome to positively impact grapevine health, growth, and grape and wine production, thereby fostering a more sustainable approach to vineyard management.

## Methods

### Experimental site

The field experiment was conducted in a vineyard located in Geisenheim, Germany (49°59’22.0"N, 7°57’00.8"E). The vineyard was planted in 1991 (*Vitis vinifera* L. cv. ‘Riesling’, clone Gm 198–30, grafted on *Vitis berlandieri* Planch. x *Vitis riparia* Michx. cv. ‘SO4’ and *Vitis riparia* Michx. x *Vitis cinerea* Engelm. cv. 'Börner’ rootstock), is 0.8 ha in size and faces south (slope <5%) .

The rows are oriented north-south and it has a row spacing of 2 m, vine spacing of 1.2 m (planting space of 2.4 m²) and is trained as a single Guyot in a vertical shoot positioning (VSP) system. The experiment was set up as a complete block design with four field replicates, each of which includes the three factor levels of the management system (Supplementary Fig. 1). Each plot consisted of four rows, with the two outermost rows serving as buffer rows between the different management systems, and samples were collected only from the two innermost rows. Each row consisted of 32 vines. A weather station located approximately 500 m from the experimental site was used for climate data collection. A summarized presentation of weather conditions throughout the experimental year 2021 is provided in Supplementary Fig. 2 [42].

### Management systems in studied area

Until the end of 2005, vineyard management adhered to Good Agricultural Practice guidelines as outlined by the European Union legislation (S.I. No. 393/2022) [43]. Commencing in 2006, selected plots within the experimental site underwent conversion to organic and biodynamic management practices in accordance with Regulation (EU) No 2018/848, as well as standards established by ECOVIN and Demeter [33, 44, 45], respectively. Meanwhile, the remaining areas continued to be managed under integrated management strategies consistent with Good Agricultural Practice principles (Table 1).

**Table 1:** Overview of the management system applied in the different treatments (modified according to [46])

	Integrated	Organic	Biodynamic
Permanent cover crop	grass mixture (every 2nd row cultivated, alternating)	multi-species mixture (every 2nd row cultivated, alternating)	
Winter cover crop	Rye, vetch	Rye, vetch or hardy multi-species mixture	
Under-vine-management	herbicides	mechanically	
Fertilization	mineral fertilizers, green waste compost	manure compost, plowing in and/or cultivation of cover crops	manure compost with biodynamic preparations, plowing in and/or cultivation of cover crops
Plant protection	systemic fungicides, mating disruption	copper (max 3 kg/ha and year), sulfur, plant strengtheners, mating disruption	
Biodynamic preparations	-	-	horn manure, horn silica, compost preparations

A multi-species mixture was used as a permanent cover crop in every other row within both organic and biodynamic plots. The present cover crop mixture on the day of sampling was sown in every second row (in-row b) on 07/29/2020 containing Wolff-mixture excluding alfalfa, whereas within the integrated treatment a grass cover (sown on 05/18/2017) was used as permanent cover crop. In all three treatments winter cover crops were sown in every other row (in-row a) on 08/27/2021, including rye and vetch for integrated plots and WB245 winter cover crops for organic and biodynamic plots. The composition of cover crop mixtures is provided (Supplementary Table 1).

Cover crops were sown according to seasonal conditions, with every second row plowed shortly before bloom in all treatments. This ensures sufficient nitrogen supply in organic and biodynamic plots by breaking up and tilling the cover crop mixture with high legume richness [46]. To compensate the nitrogen introduction in the organic and biodynamic treatments by cover crops, integrated plots were amended with mineral fertilizers (25 kg N/ha on 07/06/2006, 50 kg N/ha on 06/26/2010, 25 kg N/ha on 07/05/2012, 25 kg N/ha on 06/16/2014, 20.5 kg N/ha on 05/30/2017, and 20 kg N/ha on 07/02/2021), when soil analysis indicated a considerably reduced level of nitrogen in comparison to the organic and biodynamic plots. Since conversion, all three treatments received periodic compost applications in 2006, 2007, and 2016. Integrated plots received green waste compost, while farmyard manure sourced from organic farming was utilized for both organic and biodynamic plots, with the distinction that biodynamic compost preparations 502–507 were applied to the latter [46]. Following compost analysis, an equivalent amount of nitrogen was applied to each treatment. Under-vine weed was controlled by the application of herbicides twice a year in integrated plots, while in organic and biodynamic plots weeds were mechanically controlled. Pest management practices involved the application of systemic fungicides in the integrated plots, whereas copper, sulfur, and plant strengtheners were utilized in the organic and biodynamic plots, as detailed in Supplementary Tables 2 and 3, respectively. Both the organic and biodynamic treatments received identical soil and vine management practices, with the exception that biodynamic plots additionally received biodynamic preparations. Specifically, horn manure and horn silica, recognized as biodynamic field spray preparations, were applied three times each annually. Horn silica applications occurred at critical grapevine phenological stages, namely shortly before full-bloom, at veraison, and shortly before harvest, while horn manure was applied once post-harvest and twice during spring. If compost was not applied, the cow pat pit preparation was administered once annually during the growing season, concomitant with tillage practices [37, 47].

### Sample collection

Samples of bark (09/30/2021), leaves, and grape clusters (10/01/2021), further defined as compartments, were obtained from two randomly selected, healthy vines per row, shortly before harvest. The harvest took place on 10/12/2021. Composite samples were acquired from two vine trunks on the east side of each row. For each trunk, two sections of the outer bark, which could be peeled off, were aseptically cut and stored in sterile tubes. Similarly, composite leaf samples were obtained by collecting two leaves from each of the same two plants per row and were subsequently stored in cooled tubes. Furthermore, two clusters originating from the same two plants as the bark and leaf samples were selected from each row and placed in sterile wide-neck plastic bottles, promptly refrigerated for preservation. This sampling approach, implemented across four blocks, three management systems, and two rootstocks, yielded 24 samples for bark, leaf, and grape each. To prevent cross-contamination, the scissors used were cleaned with ethanol between sample collections. Both leaf and grape samples were washed using 0.2% Tween 80 (Carl Roth GmbH + Co. KG, Karlsruhe, Germany) with 100 ml of distilled water for leaf samples and 250 ml for grape samples. The washing process involved agitation for 1.5 h at 14 rpm using an overhead shaker (Heidolph, Schwabach, Germany). Subsequently, solid matter was separated via centrifugation at 10 °C for 30 min at 5100 rpm, and the supernatant was discarded.

### DNA extraction, PCR and sequencing

DNA extraction from bark was performed utilizing the Zymo Research Quick-DNA Fecal/Soil Microbe Miniprep Kit, while DNA from leaf and grape samples was extracted using the Zymo Research Quick-DNA™ Fecal/Soil Microbe Microprep Kit (Zymo Research Europe GmbH, Freiburg, Germany), following to the manufacturer’s instructions. Mechanical lysis of the samples was optimized through bead beating, employing Bashing Bead lysis tubes and a Disruptor precellys24 (Bertin-Instruments, Montigny-le-Bretonneux, France) at 5000 x g for 3 × 20 s. The resulting DNA was stored at − 80 °C until further processing. Quantification of DNA concentration was achieved through spectrophotometry utilizing an Epoch™ Multi-Volume Spectrophotometer System (BioTek Instruments, Vermont, USA). The fungal ITS2 region of the ribosomal DNA was amplified using the primers fITS7 and ITS4 [48] (Supplementary Table 4). Polymerase chain reaction (PCR) was conducted in 25 µl reaction volumes in duplicate, with inclusion of PCR blanks to control for potential contaminants. The standard PCR mixture comprised 12.5 µl Master Mix GoTaq Green (Promega, Wisconsin, USA), 1 µl forward primer mix (10 µM) (Biomers, Ulm, Germany), 1 µl reverse primer mix (10 µM), 9.5 µl nuclease-free water (Promega, Wisconsin, USA), and 1 µl DNA extract. The DNA template amount was adjusted based on its concentration to ensure that each reaction contained 20 ng of DNA. PCR was performed under the following thermocycler conditions: initial denaturation at 95 °C for 5 min, followed by 35 cycles of denaturation at 95 °C for 1 min, annealing at 55 °C for 1 min, extension at 72 °C for 1.5 min, and a final elongation step at 72 °C for 7 min. Amplification was carried out using a Biometra T3000 Thermocycler (LabRepCo, Pennsylvania, USA).

PCR products were separated on a 1.5% agarose gel using Power Pac 3000 Electrophoresis Power Supply (Bio-Rad Laboratories, California, USA) connected to a horizontal gel electrophoresis chamber. PCR bands were excised, and PCR products were extracted from agarose using the Wizard^®^ SV Gel and PCR Clean-Up System (Promega, Walldorf, Germany), following the manufacturer’s protocol. DNA concentrations in the purified PCR products were measured through spectrophotometry using an EpochTM Multi-Volume Spectrophotometer System (BioTek Instruments, Vermont, USA). Samples were diluted to a concentration of 10 ng/µl by the addition of nuclease-free water (Promega, Wisconsin, USA).

Gel-purified PCR products, containing Illumina Nextera XT adapter sequences to ensure compatibility with Illumina index adapters, were utilized as templates for Index PCR employing the Nextera XT Library Preparation Kit (Illumina, San Diego, CA, USA). The thermal cycling profile consisted of an initial denaturation step at 95 °C for 3 min, followed by 8 cycles of denaturation at 98 °C for 30 s, annealing at 55 °C for 30 s, and elongation at 72 °C for 30 s, with a final extension at 72 °C for 5 min. Subsequently, amplicons underwent bead purification utilizing the Agencourt AMPure XP kit (Beckman Coulter, Krefeld, Germany), followed by quantification using PicoGreen (Molecular Probes, Eugene, OR, USA). Equimolar pooling of amplicons was performed, and the resulting pool underwent a final quality assessment using an Agilent 2100 Bioanalyzer (Agilent Technologies, Palo Alto, CA, USA). This final amplicon library was subjected to 2 × 300 bp paired-end sequencing using the MiSeq Reagent kit v3 on an Illumina MiSeq system located at the Department of Soil Ecology of the Helmholtz-Centre for Environmental Research—UFZ in Halle (Saale), Germany [49].

### Raw data and bioinformatic processing

The raw data (FASTQ files) underwent processing using dadasnake (v 0.10.3) [50], which is a Linux-based Snakemake implementation of the DADA2 algorithm (v 1.14) [51], combined with several pre and post processing tools. As initial step primer sequences were identified and trimmend via cutadapt (v 4.1) [52]. Subsequently, denoising, error estimation, chimera removal, and merging were executed using DADA2. Reads exhibiting expected error rates (maxEE) exceeding 3 were excluded from further analysis. Merging was conducted with an overlap of 12 and 2 mismatches allowed. Chimera removal was carried out using the consensus algorithm.

Taxonomic classification was carried out using the mothur classifier [53] with UNITE ver. 8.2 [54] as a reference database for ITS sequences. ASVs (amplicon sequence variants) identified through differential analysis were further subjected to BLAST against the NCBI nucleotide (nt) database to verify and potentially obtain deeper taxonomic information [55].

Only sequences with a minimum length of 245 base pairs were included in the final ASV table. Quality scores for all pipeline steps were recorded by dadasnake employing fastQC (v 0.11.9) [56] and multiQC (v 1.13) [57]. All raw sequence data were submitted to NCBI SRA under BioProject number PRJNA637318 [58].

### Statistical analysis

Statistical analyses were performed using R (v 4.3) [59] and its user interface Rstudio (v 2023.06.1 + 524) [60], using the phyloseq package (v 1.16.2) to create a phyloseq object for facilitating import, storage, analysis, and graphical display of microbiome data [61]. For all statistical analysis a significance level of *p* < 0.05 was set. The number of observed ASVs (ASV richness) was calculated after rarefying the data to 10,000 sequences per sample using the phyloseq package. A linear mixed model (LMM) was applied to test whether the number of observed ASVs (hereafter referred to as fungal species richness) differed between plant compartments, management system, rootstock, and block. The model included plant compartment, management system, and rootstock as fixed effects, with block treated as a random effect to account for variability between blocks. The significance of fixed effects was evaluated using ANOVA, while the significance of the random effect (block) was assessed separately using a likelihood ratio test. Pairwise comparisons between compartments were conducted using estimated marginal means (emmeans) from the multcomp package, adjusted for multiple comparisons (v 1.4–25) [62]. Bray-Curtis dissimilarity was used to calculate the compositional similarities between samples using the phyloseq package. The homogeneity of group dispersions was assessed using the betadisper function from the vegan package (v 2.6-2) [63]. Principal Coordinates Analysis (PCoA) plots were generated to visualize the results related to plant compartments and management systems. Permutational multivariate analysis of variance (PERMANOVA) was performed to confirm ordination results. Hence, fungal community composition was tested in relation to plant compartment, management system, rootstock and block based on 999 permutations using the function adonis2 of the vegan package, followed by pairwise PERMANOVA [64]. To discern the ecological functions of fungal communities, we categorized fungal genera into functional guilds using the FungalTraits database [65]. Taxonomical and functional classification was visualized using ggplot2 by aggregating taxa at the genus level and categorizing them based on their primary lifestyle, as well as grouping samples according to compartments and management system [66]. The data were transformed to relative abundance. Genera and functional groups occurring at less than 0.5% were combined into a category labeled “Others” to enhance clarity in the plots. Differential abundance analysis was performed to identify fungal ASV representatives for the different management systems. This analysis was conducted using the ANCOMBC2 (Analysis of Composition of Microbiomes with Bias Correction 2) method, which is designed to handle the compositional nature of microbiome data and account for potential biases. The ANCOMBC2 method utilizes a log-linear model to test for differential abundance while adjusting for multiple comparisons using the Holm method [67].

## Results

The sequencing provided a total dataset of 72 samples, where 24 samples were attributed to bark, leaf and grape, respectively. For fungal data, this study generated 10,425,661 raw reads, of which 3,651,511 sequences remained after quality and chimera filter. For the remaining reads, 728 Amplicon Sequence Variants (ASV) were retained. To minimize statistical issues that could arise from differences in terms of sequencing depth, rarefaction was used to normalize the samples. Sampling depth was set at 10,000 sequences per sample and resulted in the removal of 41 ASVs.

### Fungal richness

**Fig. 1:**
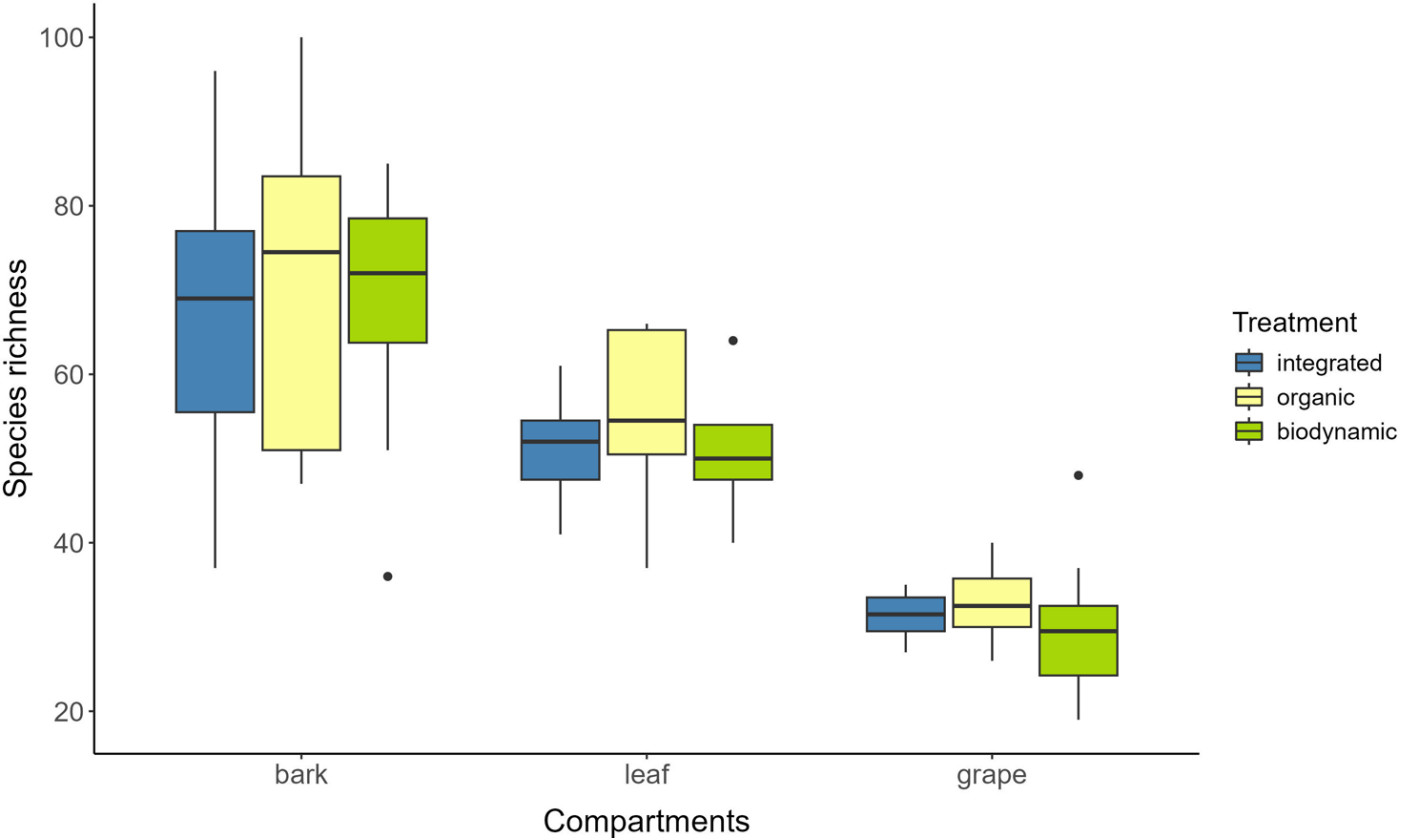
Fungal species richness by compartment and management system. The central line represents the median, boxes show the interquartile range, whiskers extend 1.5 times the interquartile range, and circles indicate outliers

Compartments exerted a significant influence on fungal species richness (*p* < 0.001) (Supplementary Table 5, Fig. 1).

The pairwise comparison revealed significant differences between all three compartments (*p* < 0.001). Bark exhibited the highest species richness, followed by leaf and grape, demonstrating mean species richness values of 68 ± 18, 52 ± 8, and 31 ± 6, respectively. These values reflect the fungal species assigned from the ASVs. In contrast, management regimes did not show a significant impact on fungal species richness (*p* > 0.05).

### Beta diversity

Based on the results of the permutational multivariate analysis of variance (PERMANOVA), significant effects of compartment, treatment, and their interaction (compartment:treatment) on fungal community composition were observed (Table 2).

**Table 2:** Results of PERMANOVA based on Bray-Curtis dissimilarity metrics for fungal community composition in relation to plant compartment, management system (treatment), rootstock and block

	Df	SumOfSqs	*R* ^2^	F	Pr(> F)
Compartment	2	10.3582	0.51894	41.6099	0.001 ***
Treatment	2	0.6542	0.03278	2.6281	0.019 *
Rootstock	1	0.1468	0.00736	1.1795	0.268 (N.S.)
Block	1	0.2171	0.01088	1.7446	0.133 (N.S.)
Compartment:treatment	4	0.9735	0.04877	1.9552	0.024 *
Compartment:rootstock	2	0.3578	0.01792	1.4372	0.172 (N.S.)
Treatment:rootstock	2	0.2978	0.01492	1.1962	0.269 (N.S.)
Compartment:treatment:rootstock	4	0.4827	0.02418	0.9694	0.421 (N.S.)
Residual	52	6.4723	0.32426	-	-
Total	70	19.9604	1.00000	-	-

*, ** and *** indicate statistical significance (*p* < 0.05, *p* < 0.01 and *p* < 0.001) of the main effects and interactions (N.S. = not significant)

Coefficients of determination indicate that 51.9% of the variation in fungal community composition is explained by compartment. Treatment and the interaction between compartment and treatment together are responsible for 8.2% variation. Pairwise multilevel comparison (pairwise PERMANOVA) revealed significant differences in beta diversity among all three compartments (*p* < 0.01) (Supplementary Table 6).

**Fig. 2:**
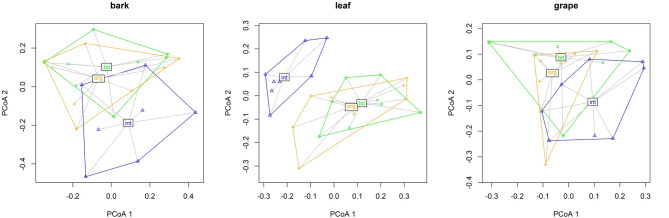
PCoA for fungal community composition based on Bray-Curtis dissimilarity within each compartment (bark, leaf, grape) displaying group centroids and dispersions in relation to management systems (int (integrated) = blue, org (organic) = yellow, bd (biodynamic) = green)

Principal Coordinates Analysis (PCoA) highlighted discrete clustering patterns for fungal community composition corresponding to management systems (Fig. 2).

A PERMANOVA model substantiated the significant dissimilarities in fungal community compositions within the compartments leaf (R²= 0.31, *p* < 0.001) and grape (R²= 0.20, *p* < 0.05), correlating with the different management systems applied. However, the fungal composition associated with bark showed no significant variation in response to the different management systems. Notably, an additional significant effect of rootstock on the fungal community composition was observed for leaf samples (R²= 0.12, *p* < 0.01) (Supplementary Table 7).

Within leaf and grape compartments, pairwise PERMANOVA analyses substantiated significant compositional dissimilarities when comparing integrated and organic systems, as well as between integrated and biodynamic systems. In contrast, the comparison between organic and biodynamic systems did not exhibit statistically significant differences (Table 3).

**Table 3:** Results of pairwise PERMANOVA for a direct comparison of differences in fungal community composition among management systems (treatment) within the subset datasets of plant compartments (leaf and grape)

	Pairs	Df	SumOfSqs	*R* ^2^	F	Pr(> F)
Leaf	integrated vs. organic	1	0.38988	0.28967	5.709	0.001 ***
	integrated vs. biodynamic	1	0.51298	0.4021	9.4151	0.001 ***
	organic vs. biodynamic	1	0.04362	0.03655	0.5311	0.792 (N.S.)
Grape	integrated vs. organic	1	0.16623	0.18018	3.0768	0.024 *
	integrated vs. biodynamic	1	0.24007	0.21253	3.7785	0.017 *
	organic vs. biodynamic	1	0.06726	0.07265	1.0967	0.359 (N.S.)

*, ** and *** indicate statistical significance (*p* < 0.05, *p* < 0.01 and *p* < 0.001) of the main effects determined by pairwise PERMANOVA (N.S. = not significant)

### Group abundances

**Fig. 3:**
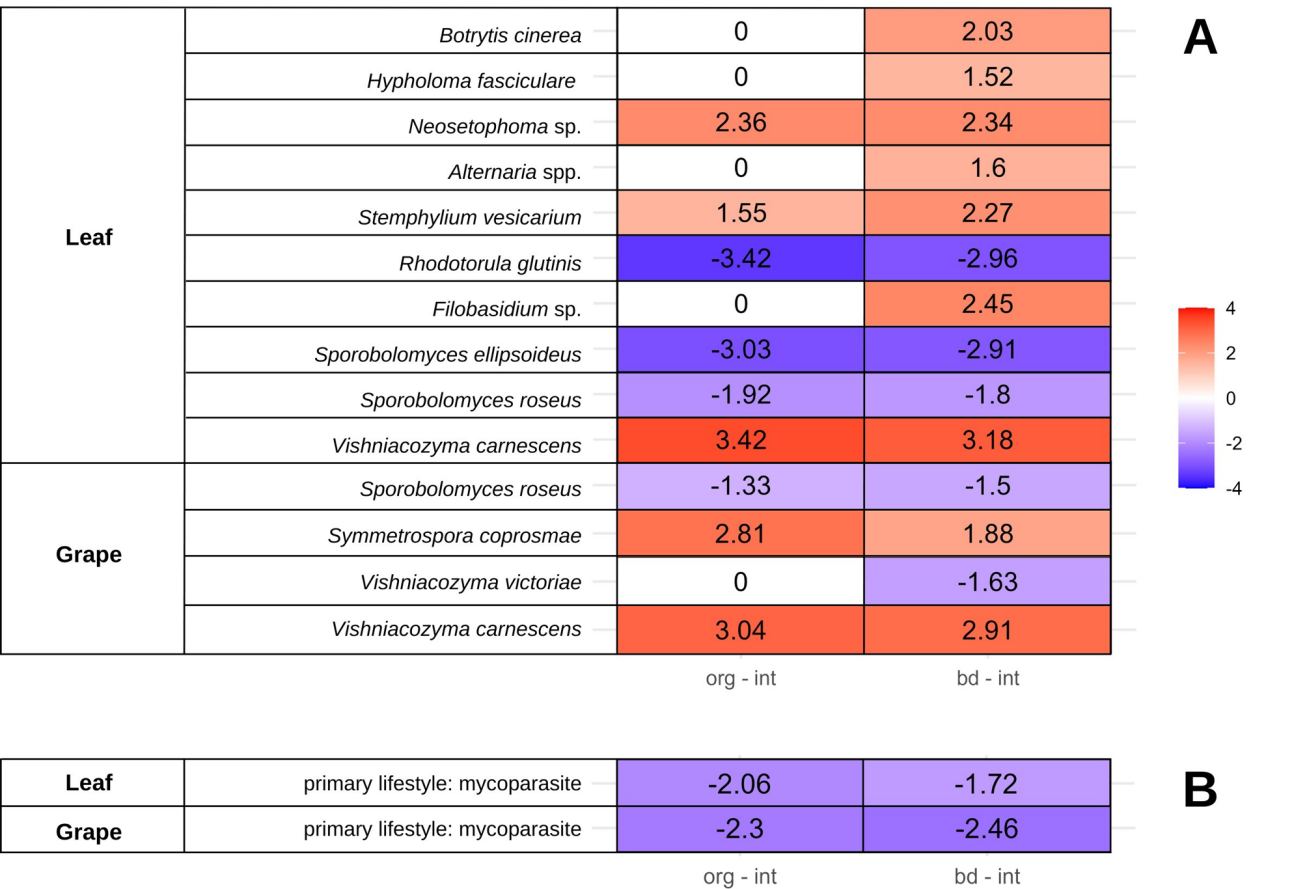
Differential abundance analysis of fungal taxa and functional groups in leaf and grape compartments, comparing organic (org) and biodynamic (bd) to integrated (int) management. (**A**) shows significant ASVs with log-fold changes, while (**B**) aggregates data by primary lifestyle categories (ANCOM-BC2, *p* < 0.05). Data annotated using FungalTraits database. Positive values (in red) indicate lower abundance in integrated management compared to organic and biodynamic management. Conversely, negative values (in purple) indicate higher abundance in integrated management

Differential abundance analyses using ANCOMBC2 were performed to explore distinct fungal taxa and functional groups between integrated and organic/biodynamic management systems (Fig. 3).

**Fig. 4:**
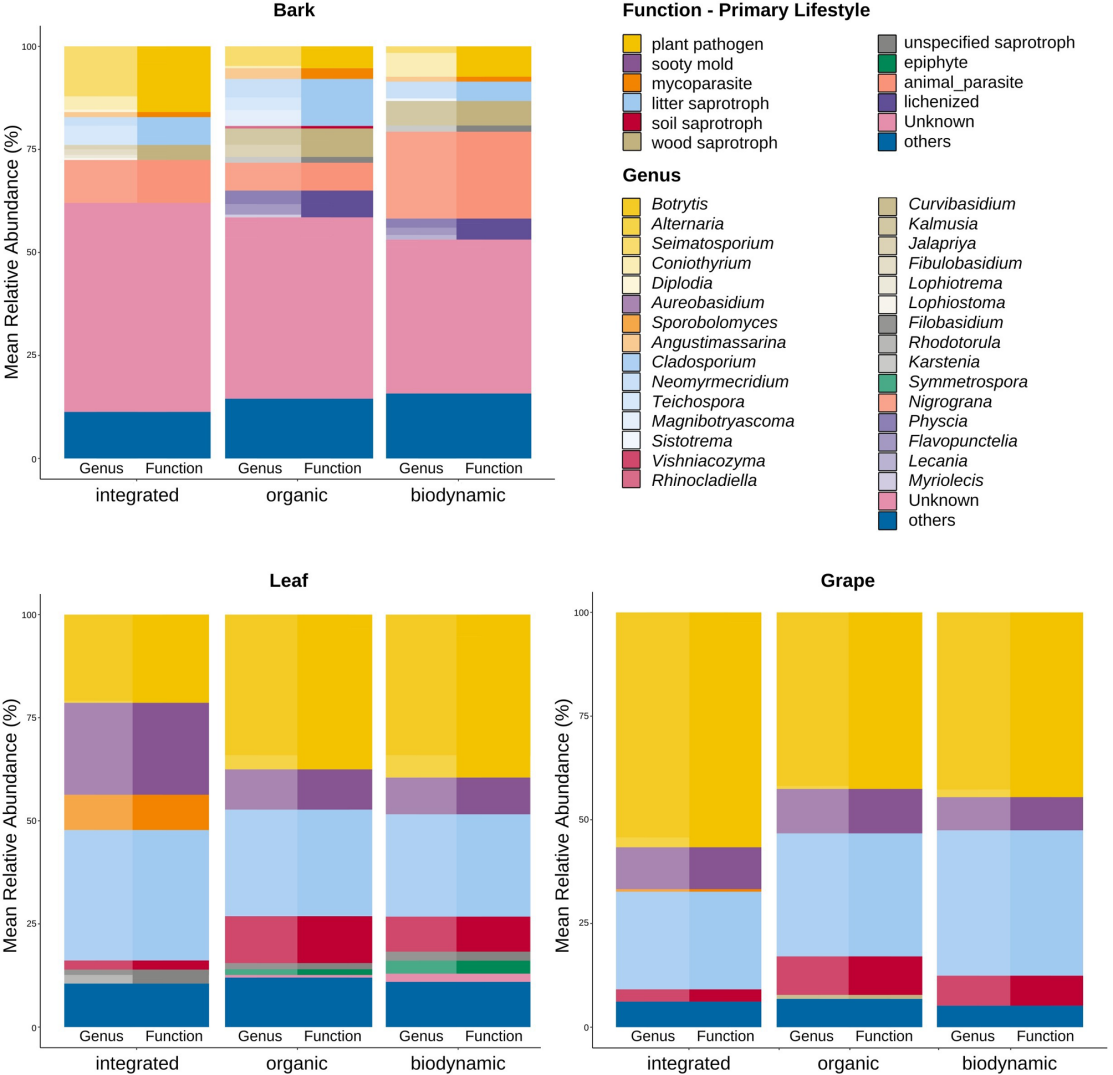
Proportions of fungal genera (left) and their primary lifestyle according to FungalTraits database (right) on bark, leaf and grape according to management system based on mean relative abundance of ASVs (%)

Taxonomic classifications at the ASV level were labeled according to the closest identifiable taxonomic rank. The comparison between organic and biodynamic treatments did not reveal significant differences in community composition; consequently, no differential analysis was conducted for these groups. Similarly, no significant treatment effects on community composition were detected for bark, and therefore, differential analysis was not applied to this compartment either. The bark compartment exhibited dominance by fungal ASVs that could not be identified up the species level using the UNITE database. The taxonomic composition according to the mean relative abundance of the leaf and grape compartments appeared more similar to each other compared to the bark compartment, showing fungal communities predominated by plant pathogens, particularly *Botrytis cinerea* and *Alternaria* spp. (Fig. 4).

In the leaf compartment, mean relative abundance suggested a higher relative abundance of plant pathogens in the organic and biodynamic treatments compared to integrated management. However, differential analysis revealed no significant differences at the functional group level for pathogens (Fig. 3).

On a finer scale, significant variations were detected at the ASV level. Here, both *Botrytis cinerea* and *Alternaria* spp. exhibited a statistically higher abundance under biodynamic management when contrasted with integrated practices. In the context of organic management, this trend was not statistically supported, as indicated by log fold changes denoted as zero, highlighting the absence of significant differential abundance for *Botrytis cinerea* and *Alternaria* spp. between organic and integrated management systems. Additionally, *Stemphylium vesicarium*, which also belongs to the functional groups of pathogens, was significantly higher within the organic and biodynamic treatment when compared to the integrated system.

Conversely, for the grape compartment, the mean relative abundance suggested an inverse trend, indicating a higher prevalence of pathogens, mainly *Botrytis* and *Alternaria* in the integrated treatment compared to the organic and biodynamic systems (Fig. 4). However, this observation was not supported by statistically significant differences in the differential analysis (Fig. 3).

In the grape and leaf compartments, a lower abundance of mycoparasites was observed in the organic and biodynamic treatments compared to the integrated management at the functional level. Differential abundance analysis at the ASV level revealed a decreased presence of *Sporobolomyces roseus* in both grape and leaf compartments under organic and biodynamic management. Additionally, *Sporobolomyces ellipsoideus* and *Rhodotorula glutinis* exhibited lower abundance in the leaf compartment.

Moreover, certain species displayed varying abundance patterns across different management systems, all within the broader category of saprotrophs. However, no consistent trend emerged, indicating that no single management system consistently supported a higher abundance of saprotrophs overall.

## Discussion

As the agricultural sector moves towards sustainability, driven by environmental concerns and initiatives like the European Green Deal, there is an imperative need to understand how management practices influence microbial communities. This understanding is crucial for devising farming strategies that balance beneficial and pathogenic microbial communities, thereby fostering more resilient agricultural systems that align with the goals of sustainability [1]. With this study, we aim to contribute to the growing body of knowledge on sustainable agricultural practices by comparing fluctuations in above-ground fungal community composition and changes in functional diversity caused by different viticultural management systems (integrated, organic and biodynamic).

In terms of alpha diversity, our study did not detect any significant variances in species richness among different management systems, irrespective of the corresponding compartments. This can be placed into context with results from previous studies, stating no differences in fungal richness among management systems within various grapevine compartments [26, 27, 29, 35, 68–71]. However, it is noteworthy that contradictory outcomes have been reported. Some studies have documented higher richness or enzymatic activity (β-glucosidase and urease) associated with organic/biodynamic management in comparison to conventional treatments [28, 72, 73]. Nonetheless, the general range of fungal richness observed in this study is consistent with findings from similar studies [35, 69, 74, 75].

Although fungal community composition (beta diversity) was primarily influenced by compartments, the impact of management system, as well as the interaction between management system and compartments, also significantly influenced community composition. An interaction effect was evident, as the management systems did not uniformly influence fungal communities across all compartments. Specifically, communities on bark appeared unaffected, whereas significant management effects were observed for leaf and grape compartments. This observation is consistent with prior research comparing fungal communities in conventionally and biodynamically managed vineyards, which found no discernible differences in fungal community composition on trunks [26]. This phenomenon could be attributed to management practices primarily targeting green tissues with distinct plant protection measures, potentially limiting direct effects on the vine’s trunk area. It is also worth considering how fungal communities might differ in spur-pruned and cordon-trained vineyards, where the perennial wood structures are exposed to fungicide applications over multiple years. The fungal communities in cordon-trained systems may be more similar to those of the trunk due to the perennial nature of the cordons, but could also be influenced by direct fungicide applications. Comparisons between cane- and spur-pruned woods would provide valuable insights into how these fungicide applications influence fungal communities in woody structures.

Moreover, leaves and grapes can be categorized as annual structures undergoing differentiation over one vegetative cycle, whereas bark represents a perennial structure. It can be hypothesized that the microbiota associated with perennial woody parts experience less variation compared to microbiota of other aerial parts due to its permanence [40]. Additionally, it is important to note that the bark tissue sampled in this study was scraped off the trunk, meaning it consisted primarily of non-living tissue, in contrast to the actively growing leaves and fruit. This difference in tissue vitality may further contribute to the distinct fungal communities observed across compartments. These hypotheses align with the findings regarding fungal species richness among compartments, with bark exhibiting the highest richness followed by leaf and grape, suggesting that bark harbors greater diversity due to its stability over time. Taxonomic composition revealed a high diversity within the bark compartment, characterized by a broad array of fungal species, with a notable percentage of unidentified taxa derived from the UNITE database. The presence of numerous unknown species poses a challenge in accurately assessing the impact of management practices on fungal communities, as these unknown taxa may obscure the management effect. This underscores the complexity of fungal ecology and the limitations in fully capturing the diversity of microbial communities due to incomplete reference databases.

A notable disparity in the structure of fungal communities on leaves and grapes was observed, influenced by the type of viticultural management. This suggests that while the overall diversity may remain similar, the composition and abundance of species are affected by management practices. These findings align with previous studies, indicating differences in community structure induced by various management practices [26, 68–70, 72, 76–79]. Specifically, a distinct shift in fungal community composition was observed between integrated and organic/biodynamic treatments, yet no discernible difference between organic and biodynamic fungal communities was evident. Consequently, the additional application of biodynamic preparations did not induce a change in fungal community composition in the current investigation. This finding is in line with several previous studies that have reached similar conclusions [25, 27, 28, 79]. In addition to the effects of management systems on fungal communities, this study also observed a significant rootstock effect in the leaf compartment. This finding can be attributed to the influence of rootstocks on the physiological traits of the scion, which can alter vegetative parameters such as leaf area and canopy development [80]. These changes in foliar conditions may, in turn, induce shifts in fungal community composition [81].

While the understanding of the structural composition lays a foundation, it’s the functional connection to abundant taxa that may ultimately provide the most promising avenues for advancing sustainable agriculture. However, structural and functional differences must be considered equally to understand which taxa are responsible for functional disparities.

Generally, the fungal communities on leaves and grapes were dominated by plant pathogens, notably *Botrytis cinerea* and *Alternaria* spp. *Botrytis cinerea* is a necrotrophic fungal pathogen causing grape bunch rot and gray mold, known to produce off-flavors such as earthy or moldy odors in grape juice and wines [81–83]. It can lead to significant economic losses in vineyards worldwide, particularly in conditions of continuous mild wet weather [84]. However, in specific microclimatic conditions, such as humid nights followed by dry sunny days, *Botrytis cinerea* can induce noble rot, which is highly sought after for the production of botrytized wines [85]. Thus, while *Botrytis cinerea* is generally regarded as a destructive pathogen in grape production, its classification as beneficial or pathogenic can depend on production goals and the desired wine style. *Alternaria*, a filamentous fungus, is frequently identified as a predominant organism within the grapevine phyllosphere [20, 70, 86, 87]. While its presence can contribute to yield losses and modifications in chemical composition, it is generally considered less economically significant compared to major pathogens like *Botrytis cinerea*. However, certain *Alternaria* strains are capable of producing mycotoxins, which can pose a potential risk to wine consumers [88]. It was expected to observe a high abundance of mold-inducing pathogens, as the year 2021 was characterized by high temperatures and several significant precipitation peaks, particularly in the mid-summer months (Supplementary Fig. 2). The combination of elevated moisture levels and warmer temperatures typically creates ideal conditions for the growth and spread of fungal pathogens [89].

In the leaf compartment, relative taxonomic analysis revealed an increase in plant pathogens under organic and biodynamic treatments compared to integrated management. Notably, the abundance of ASVs in organic and biodynamic treatments followed similar trends, suggesting comparable effects from these management practices. However, significant differences were detected only at the ASV level, with *Botrytis cinerea* and *Alternaria* spp. exhibiting higher abundance under biodynamic management compared to integrated practices. This pattern was not statistically significant for organic management, likely due to the stringent nature of the differential analysis conducted by ANCOMBC2. Given the absence of differences in community composition (beta diversity) between organic and biodynamic management, these findings imply that the observed discrepancies may be attributable to statistical limitations rather than real differences between organic and biodynamic practices.

Interestingly, in the grape compartment, an inverse trend is observed, indicating a higher prevalence of pathogens in the integrated treatment compared to organic and biodynamic management systems, although no statistically significant differences were detected upon differential analysis. This result suggests that while management practices may influence the presence of pathogens, the direct correlation to their abundance on grapes could not be conclusively established through the available data.

A notable discrepancy in fungal community composition on leaves and grapes among different management systems has been identified, particularly in relation to mycoparasites. Integrated management systems exhibit higher abundances of *Sporobolomyces roseus*, *Sporobolomyces ellipsoideus*, and *Rhodotorula glutinis*. Notably, *Sporobolomyces roseus* and *Rhodotorula glutinis* have garnered attention as potential biocontrol agents due to their antagonistic behavior against *Botrytis cinerea* and *Penicillium expansum* in various fruits. These species suppress fungal growth primarily through competition for space and nutrients, thereby directly impacting disease incidence and severity [90–96].

Consistent findings have been reported in other studies, which also observed higher abundances of species belonging to the order *Sporidiobolales* in conventional vineyards, identifying them as indicator species [76, 97]. It is conceivable that copper or sulfur treatments may disproportionately affect *Sporidiobolus* species, leading to decreased abundance under organic and biodynamic management [98].

While the genus *Alternaria* is commonly recognized for its pathogenic associations with various crops [99, 100], it is essential to note that this genus encompasses numerous species that function as saprophytes and endophytes [69, 101, 102]. Moreover, certain species within this genus exhibit beneficial effects, such as antagonistic properties against *Plasmopara viticola* and *Botrytis cinerea* [103, 104], underscoring their potential as biocontrol agents.

This highlights the limitations of functional prediction using the FungalTraits Database, as the genus *Alternaria* is primarily categorized as pathogen. Ecological guilds, which represent groups performing similar functional roles in an ecological community, are valuable for inferring and predicting the functional characteristics of microbial communities [83]. However, the linkage of lifestyle at the genus level partly fails to capture the entire spectrum of ecological functions, as species within a genus may serve diverse roles in ecosystems [65]. This highlights the need for functional prediction at the species level to increase the resolution of functional information.

As a whole it can be stated that the fungal community composition was shown to be affected by management systems, with integrated communities being significantly distinct from organic and biodynamic communities. These observations may be attributed to the application of synthetic or organic pesticides, which directly impact microorganisms, or to alterations in plant physiology, subsequently affecting plant-associated microorganisms.

The impact of various spraying regimes on fungal community composition within agricultural management systems remains a contentious issue in the current discourse. Copper, as a fungicide, generally exhibits a broader spectrum of activity compared to synthetic molecules commonly employed in integrated management approaches [105]. Notably, microbial communities sensitive to copper appear to be particularly susceptible to alterations. Elevated copper concentrations resulted in a negative correlation with yeast and yeast-like populations on above-ground tissues, providing compelling evidence of copper-induced shifts in epiphytic communities [76]. Moreover, the prevalence of the genus *Sporidiobolus*, categorized as mycoparasites, within integrated treatment systems is noteworthy. This phenomenon may be attributed in part to the inherent resistance of this genus to synthetic fungicides [106] and its susceptibility to copper [98], thereby highlighting the premise that disparities in spraying regimes within management systems promoting a dominance of resilient species. However, several studies comparing microbial communities on above-ground tissues subjected to varying pesticides have yielded intriguing results. The comparisons between copper and bioagent treatments, various systemic fungicides, and chemical pesticides versus biocontrol agents failed to reveal significant differences in community composition [83, 107, 108]. These findings suggest that observed changes in fungal community composition may not solely be attributable to differing spraying regimes. Instead, they imply that additional factors beyond pesticide selection might influence the composition of above-ground tissue communities within the prevailing management systems.

Welsh et al. [79] observed minimal differences in diversity and species richness between copper and sulfur-based fungicide groups and a water control. They noted a consistent decline in diversity and richness across all treatments, likely due to the washing effect of applying treatments. These findings suggest that the frequency of application may play a more significant role than the specific type of pesticide in driving changes within community dynamics among our management systems. This effect may be particularly relevant in temperate climates, where frequent rainfall requires repeated applications, especially in organic and biodynamic systems. In warmer regions with less rainfall, the frequency of copper and sulfur applications may be reduced, potentially lessening the impact of the washing effect and highlighting differences in fungicides used across management systems. Nevertheless, it is imperative to broaden our understanding of pesticide effects beyond their impact on pathogenic microorganisms. While considerable attention has been directed towards this aspect, the potential influence on beneficial microorganisms should not be overlooked. Prioritizing the understanding and promotion of a self-regulating microbiome through targeted spraying practices, rather than indiscriminate suppression, is imperative.

In addition to differences in spraying regimes, alterations in plant physiology induced by different management systems could offer another explanation for changes in fungal community composition on leaves and grapes. Studies show that organic and biodynamic practices reduce leaf area, lateral shoot growth, shoot length and pruning weight, despite better nitrogen availability [46, 109, 110]. Furthermore, a consistent yield reduction under organic and biodynamic management could be observed, with notably less bunch compactness, lower number of berries per bunch and reduced average bunch weights compared to integrated approaches [111–115]. Döring et al. [46]. presented compelling evidence indicating disparities in vine water status and physiological performance between organic or biodynamic and conventional viticulture practices. This may be explained by the use of cover crops, which are characteristic of organic and biodynamic systems to enhance biodiversity and nitrogen supply, as their presence can reduce soil water availability and instigate competition for both water and nutrients [116]. These divergences in physiological performance could also potentially drive alterations in fungal community structure by indirectly inducing shifts in compartment conditions (i.e. canopy microclimate), thereby favoring different microbial communities.

## Conclusion

The study’s findings underscore the influence of management practices on fungal community dynamics across three above-ground plant compartments.

Advances in microbial ecology, facilitated by technological improvements in molecular biology and genomics, have enabled detailed analysis of microorganism diversity. These developments have not only broadened our understanding but also highlighted the complexities involved in converting microbial data into actionable agricultural insights. Consequently, future investigations should prioritize examining individual agricultural practices, such as spraying regimens or foliage management techniques, rather than entire systems. This focused approach is crucial for simplifying the complex interactions between microorganisms and their environments, uncovering the precise drivers behind shifts in community structure, and revealing strategies to naturally modulate the microbiome, thereby facilitating the proliferation of beneficial microorganisms. Nevertheless, our research highlights the potential of targeted management to strategically influence fungal communities above-ground, potentially improving plant health and increasing crop yields in line with sustainable agricultural objectives. Looking ahead, it will be essential to incorporate more detailed functional analyses to deepen our understanding of the ecological roles of these microbial communities. Such comprehensive insights may lead to the development of innovative management strategies that promote vineyard health and enhance sustainability.

## Electronic supplementary material

Below is the link to the electronic supplementary material.

Supplementary Material 1

Supplementary Material 2

## Data Availability

The data supporting the findings of this study are included within the paper and its Supplementary Information files. All raw sequence data were submitted to NCBI SRA under BioProject number PRJNA637318.
