# Supplementary material for: Functional diversity of the above-ground fungal community under long-term integrated, organic and biodynamic Vineyard Management
Source: Environ Microbiome. 2024 Nov 11;19:89. doi: 10.1186/s40793-024-00625-x (PMC11575106; doi:10.1186/s40793-024-00625-x)
Supplement: Supplementary file 2 — Supplementary Material 2 [file 40793_2024_625_MOESM2_ESM.pdf]

Supplementary Figures and Tables for:

Functional Diversity of the above-ground Fungal Community under long-term integrated, organic and biodynamic vineyard management

Katharina Steng[1], Friederike Roy[2], Harald Kellner[2], Julia Moll[3], Susanne Tittmann[1], Johanna Frotscher[4], Johanna Döring[1]

[1]Department of General and Organic Viticulture, Hochschule Geisenheim University, Von-Lade-Str. 1, 65366 Geisenheim, Germany

[2]Department of Bio- and Environmental Sciences, TU Dresden, International Institute Zittau, Markt 23, 02763 Zittau, Germany

[3]Department of Soil Ecology, Helmholtz Centre for Environmental Research GmbH - UFZ, Theodor-Lieser-Str. 4, 06120 Halle, Germany

[4]Department of Grapevine Breeding, Hochschule Geisenheim University, Von-Lade-Str. 1, 65366 Geisenheim, Germany

Contents

Supplementary Figure 1: Graphic depiction of the experimental site 1

Supplementary Figure 2: Overview of weather conditions throughout the experimental year 2021. Weather data collected from Weather station “Mäuerchen” located in Geisenheim, Germany (HSG - Vineyard protection, viewed on 09/15/2022). 1

Supplementary Table 1: Composition of the different types of cover crops present in studied area according to treatment. 2

Supplementary Table 2: Overview of integrated pest management in 2021. 3

Supplementary Table 3: Overview of organic and biodynamic pest management in 2021. 4

Supplementary Table 4: Primers used in this study. P5 and P7 are the Illumina overhang adapter sequences to allow binding during Index PCR and finally to the flow cell. N is the number of random nucleotides included between the target primers (fITS7 / ITS4) and Illumina adapter to increase the diversity of generated amplicons and thus the quality of sequencing results. 5

Supplementary Table 5: Results of the linear mixed model (LMM) analysis for fungal species richness, with compartment, treatment, and rootstock as fixed effects, and block as a random effect. ANOVA was used to determine the significance of the fixed effects and their interactions, while a likelihood ratio test was used to evaluate the random effect (block). *, **, and *** indicate statistical significance (p<0.05, p<0.01, and p<0.001), while N.S. represents non-significant results. 6

Supplementary Table 6: Results of pairwise PERMANOVA for a direct comparison of differences in fungal community composition among plant compartments. *, ** and *** indicate statistical significance (p<0.05, p<0.01 and p<0.001) of the main effects determined by pairwise PERMANOVA (N.S. = not significant). 6

Supplementary Table 7: Results of PERMANOVA based on Bray-Curtis dissimilarity metrics for fungal community composition within the data subsets of each plant compartment in relation to management system (treatment), rootstock and block. *, ** and *** indicate statistical significance (p<0.05, p<0.01 and p<0.001) of the main effects, (N.S. = not significant). 7

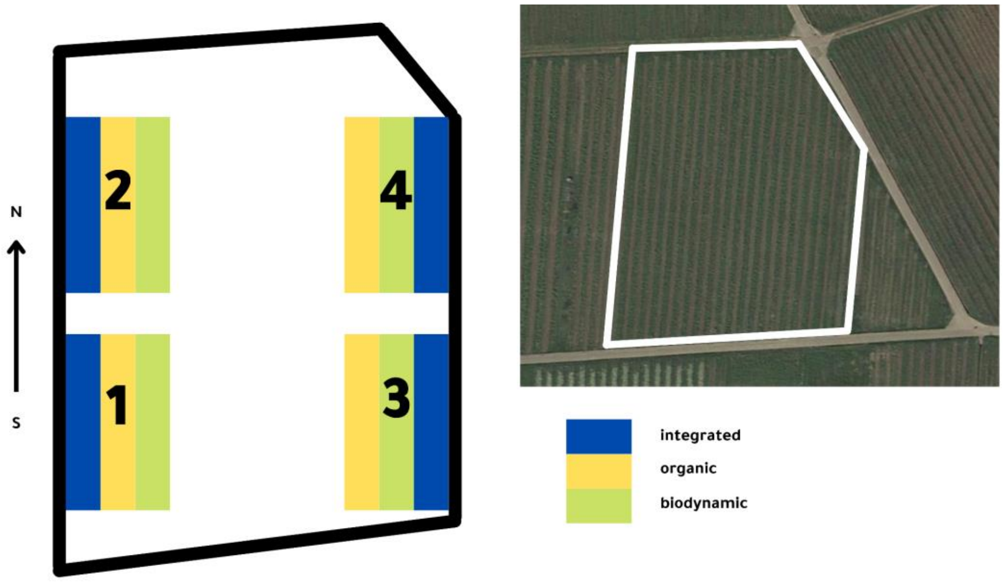

Supplementary Figure 1: Graphic depiction of the experimental site

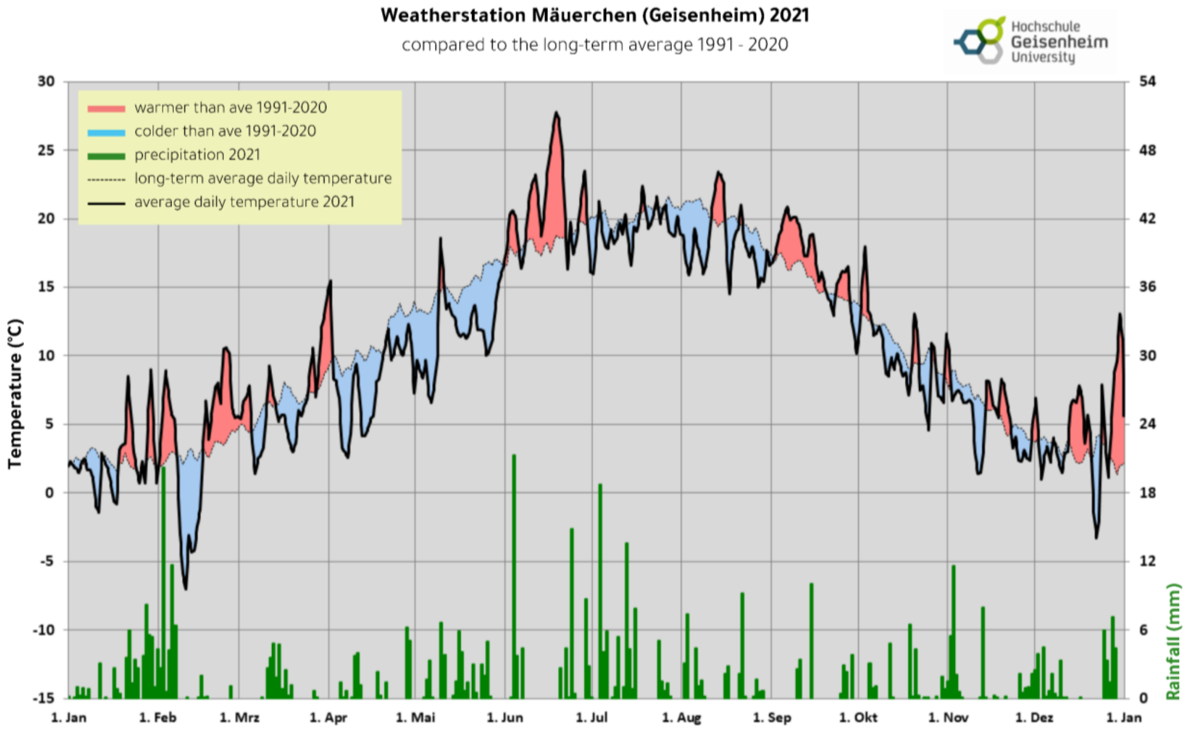

Supplementary Figure 2: Overview of weather conditions throughout the experimental year 2021. Weather data collected from Weather station "Mäuerchen" located in Geisenheim, Germany (HSG - Vineyard protection, viewed on 09/15/2022).

Supplementary Table 1: Composition of the different types of cover crops present in studied area according to treatment.

Treatment	Type of cover crop	Name	Composition		Percentage
integrated	permanent cover crop	Mulch mixture II (Freudenberger, seeds WB120)	*Poa pratensis*		80%
			Lolium perenne		20%
	winter cover crop	Vetch/Rye	*Secale cereale*		80%
			Vicia sativa		20%
organic & biodynamic	permanent cover crop	Wolff-mixture without Lucerne	*Trifolium alexandrinum*		8.1%
			Trifolium incarnatum		8.1%
			Phacelia tanacetifolia		2.7%
			Lathyrus latifolius		21.6%
			Melilotus albus		8.1%
			Onobrychis spec.		16.2%
			Medicago lupulina		5.4%
			Trifolium resupinatum		5.4%
			Trifolium hybridum		2.7%
			"Bienenweidenmischung":	*Phacelia spec.*	10.8%
				Fagopyrum esculentum	
				Coriandrum sativum	
				Calendula officinalis	
				Nigella sativa	
				Raphanus sativus var. Oleiformis Pers.	
				Malva sylvestris	
				Borago officinalis	
				Anethum graveolens	
				Helianthus annuus	
			"Würzkräutermischung"	*Sanguisorba minor*	10.8%
				Carum carvi	
				Plantago lanceolata	
				Cichorium intybus	
				Achillea millefolium	
				Daucus carota subsp. carota	
				Petroselinum crispum	
				Foeniculum vulgare	
				Pastinaca sativa	
				Lotus corniculatus	
	winter cover crop	winter cover crop mixture (WB245) 70% organic	*Trifolium incarnatum*		10%
			Phacelia tanacetifolia		3%
			Secale cereale		45%
			Brassica rapa		4%
			Vicia sativa		30%
			"Würzkräutermischung"	*Sanguisorba minor*	8%
				Carum carvi	
				Plantago lanceolata	
				Cichorium intybus	
				Achillea millefolium	
				Daucus carota subsp. carota	
				Petroselinum crispum	
				Foeniculum vulgare	
				Pastinaca sativa	
				Lotus corniculatus	

Supplementary Table 2: Overview of integrated pest management in 2021.

date	agent	active substance	quantity of agent	unit
04/09/21	RAK 1+2 Geisenheim		500	dispensers ha^{-1}
04/10/21	Folpan	Folpet	0.4	kg ha^{-1}
05/26/21	wettable sulfur	sulfur	5	Ltr ha^{-1}
05/27/21	Zorvec	Mancozeb, Oxathiapiprolin	0.16	Ltr ha^{-2}
06/10/21	Flovine	Folpet	0.8	kg ha^{-1}
	Talendo	Proquinazid	0.2	Ltr ha^{-2}
	wettable sulfur	sulfur	1.5	kg ha^{-1}
	Delan Pro	Kaliumphosphonat, Dithianon	3	kg ha^{-1}
06/24/21	Luna Experience	Tebuconazol, Fluopyram	0.313	Ltr ha^{-2}
	Ampexio	Zoxamide Mandipropamid	0.48	kg ha^{-1}
07/08/21	Dynali	Difenoconazol	0.8	Ltr ha^{-2}
07/09/21	Orvego	Ametoctradin, Dimethomorph	1.6	Ltr ha^{-2}
07/20/21	Sercadis	Fluxapyroxad	0.24	Ltr ha^{-2}
07/21/21	Delan Pro	Kaliumphosphonat, Dithianon	3.6	kg ha^{-1}
08/03/21	Vivando	Metrafenone	0.32	Ltr ha^{-2}
08/04/21	Lebosol Mg 400Sc		2	kg ha^{-1}
	Folpan 80 WDG	Folpet	1.6	Ltr ha^{-2}
08/17/21	Systhane 20 EW	Myclobutanil	0.24	kg ha^{-1}
	Lebosol Mg 400Sc		2	kg ha^{-1}
08/20/21	Kumar	Potassiumhydrogencarbonate	5	kg ha^{-1}

Supplementary Table 3: Overview of organic and biodynamic pest management in 2021.

date	agent	quantity of agent	unit	amount of copper [g ha^{-1}]
04/09/21	RAK 1+2 Geisenheim	500	dispensers ha^{-1}	
05/26/21	wettable sulfur	3,6	kg ha^{-1}	
	Funguran Progrss (100 g Cu/ha)	0.286	kg ha^{-1}	100
	Water	150	Ltr ha^{-1}	
06/08/21	wettable sulfur	4	kg ha^{-1}	
	Funguran Progress (200 g Cu/ha)	0.857	kg ha^{-1}	200
	Water	270	Ltr ha^{-1}	
06/17/21	wettable sulfur	5	kg ha^{-1}	
	Funguran Progress (200 g Cu/ha)	1.429	kg ha^{-1}	200
	Water	360	Ltr ha^{-1}	
06/23/21	wettable sulfur	4	kg ha^{-1}	
	Funguran Progress (200 g Cu/ha)	1.143	kg ha^{-1}	200
	Water	500	Ltr ha^{-1}	
06/30/21	wettable sulfur	4	kg ha^{-1}	
	Funguran Progress (400 g Cu/ha)	1.143	kg ha^{-1}	400
	Water	450	Ltr ha^{-1}	
07/08/21	wettable sulfur	5	kg ha^{-1}	
	Funguran Progress (400 g Cu/ha)	1.143	kg ha^{-1}	400
	VitiSan (3,0 Kg/ha)	3	kg ha^{-1}	
	WetCid (0,2%)			
	Water	533	Ltr ha^{-1}	
07/15/21	wettable sulfur	5	kg ha^{-1}	
	Funguran Progress (300 g Cu/ha)	0.857	kg ha^{-1}	300
	VitiSan (3,0 Kg/ha)	3	kg ha^{-1}	
	WetCid (0,2%)			
	Water	533	Ltr ha^{-1}	
07/23/21	wettable sulfur	4	kg ha^{-1}	
	Funguran Progress (300 g Cu/ha)	0,857	kg ha^{-1}	300
	Vitisan (4 kg/ha)	4	kg ha^{-1}	
	Wetcit (0,2%)			
	Water	533	Ltr ha^{-1}	
07/30/21	Funguran Progress (300 g Cu/ha)	0.857	kg ha^{-1}	300
	Vitisan (5 kg/ha)	5	kg ha^{-1}	
	Wetcit (0,2%)			
	Water	600	Ltr ha^{-1}	
08/06/21	Vitisan 6,0 kg/ha	6	kg ha^{-1}	
	Profital fluid (0,15%)			
	Water	600	Ltr ha^{-1}	
	Copranol Duo (300 g Cu/ha)	1.074	kg ha^{-1}	300
08/16/21	Vitisan 6,0 kg/ha	6	kg ha^{-1}	
	Profital fluid (0,15%)			
	Water	600	Ltr ha^{-1}	
	Copranol Duo (300 g Cu/ha)	1,074	kg ha^{-1}	300
08/20/21	Kumar	5	kg ha^{-1}	**Total**
				3000

Supplementary Table 4: Primers used in this study. P5 and P7 are the Illumina overhang adapter sequences to allow binding during Index PCR and finally to the flow cell. N is the number of random nucleotides included between the target primers (fITS7 / ITS4) and Illumina adapter to increase the diversity of generated amplicons and thus the quality of sequencing results.

ITS2 primer	primer name	primer sequence
forward	P7_X3N_fITS7	5'- GTC TCG TGG GCT CGG AGA TGT GTA TAA GAG ACA GNN NGT GAR TCA TCG AAT CTT TG-3'
	P7_X4N_fITS7	5'-GTC TCG TGG GCT CGG AGA TGT GTA TAA GAG ACA GNN NNG TGA RTC ATC GAA TCT TTG-3'
reverse	P5_X5N_ITS4	5'-TCG TCG GCA GCG TCA GAT GTG TAT AAG AGA CAG NNN NNT CCT CCG CTT ATT GAT ATG C-3'
	P5_X6N_ITS4	5'-TCG TCG GCA TCA GAT GTG TAT AAG AGA CAG NNN NNN TCC TCC GCT TAT TGA TAT GC-3'

*Supplementary Table 5: Results of the linear mixed model (LMM) analysis for fungal species richness, with compartment, treatment, and rootstock as fixed effects, and block as a random effect. ANOVA was used to determine the significance of the fixed effects and their interactions, while a likelihood ratio test was used to evaluate the random effect (block). *, **, and *** indicate statistical significance (p<0.05, p<0.01, and p<0.001), while N.S. represents non-significant results.*

**Fixed effects**	**Df**	**Sum Sq**	**Mean Sq**	**F value**	**P value**
compartment	2	15853.8	7926.9	57.4951	<0.001 ***
treatment	2	188.9	94.5	0.6851	0.5087 (N.S.)
rootstock	1	29.8	29.8	0.2158	0.6443 (N.S.)
compartment:treatment	4	38.3	9.6	0.0695	0.9909 (N.S.)
compartment:rootstock	2	76.2	38.1	0.2764	0.7596 (N.S.)
treatment:rootstock	2	292.1	146.0	1.0593	0.3543 (N.S.)
compartment:treatment:rootstock	4	480.2	120.1	0.8708	0.4881 (N.S.)
**Random effect**	**Df**	**logLik**	**AIC**	**LRT**	**P value**
block	1	-221.42	480.84	3.0421	0.08113 (N.S.)

*Supplementary Table 6: Results of pairwise PERMANOVA for a direct comparison of differences in fungal community composition among plant compartments. *, ** and *** indicate statistical significance (p<0.05, p<0.01 and p<0.001) of the main effects determined by pairwise PERMANOVA (N.S. = not significant).*

**Pairs**	**Df**	**SumOfSqs**	**R^2**	**F**	**Pr(>F)**
bark vs leaf	1	7.4525	0.48122	41.742	0.001 ***
bark vs grape	1	7.7200	0.506	46.093	0.001 ***
leaf vs grape	1	0.4651	0.11349	5.8889	0.002 **

*Supplementary Table 7: Results of PERMANOVA based on Bray-Curtis dissimilarity metrics for fungal community composition within the data subsets of each plant compartment in relation to management system (treatment), rootstock and block. *, ** and *** indicate statistical significance (p<0.05, p<0.01 and p<0.001) of the main effects, (N.S. = not significant).*

		**Df**	**SumOfSqs**	**R^2**	**F**	**Pr(>F)**
bark	treatment	2	0.6812	0.11413	1.3173	0.120 (N.S.)
	rootstock	1	0.2054	0.03441	0.7943	0.691 (N.S.)
	block	1	0.4277	0.07166	1.6542	0.038 *
	Residual	18	4.6545	0.77979	-	-
	Total	22	5.9689	1.00000	-	-
leaf	treatment	2	0.63098	0.30551	5.4403	0.001 ***
	rootstock	1	0.24030	0.11635	4.1437	0.007 **
	block	1	0.09221	0.04465	1.5900	0.176 (N.S.)
	Residual	19	1.10184	0.53349	-	-
	Total	23	2.06532	1.00000	-	-
grape	treatment	2	0.31571	0.20135	2.7067	0.016 *
	rootstock	1	0.05715	0.03645	0.9799	0.410 (N.S.)
	block	1	0.08702	0.05550	1.4921	0.195 (N.S.)
	Residual	19	1.10808	0.70670	-	-
	Total	23	1.56796	1.00000	-	-
